# Dosimetric evaluation of Plastic Water Diagnostic Therapy

**DOI:** 10.1120/jacmp.v9i2.2761

**Published:** 2008-04-29

**Authors:** Ramani Ramaseshan, Kirpal Kohli, Fred Cao, Robert Heaton

**Affiliations:** ^1^ Medical Physics Department BC Cancer Agency, Fraser Valley Centre Surrey British Columbia; ^2^ Radiation Physics Princess Margaret Hospital Toronto Ontario Canada

**Keywords:** Radiation therapy, phantoms, Plastic Water

## Abstract

High‐precision radiotherapy planning and quality assurance require accurate dosimetric and geometric phantom measurements. Phantom design requires materials with mechanical strength and resilience, and dosimetric properties close to those of water over diagnostic and therapeutic ranges. Plastic Water Diagnostic Therapy (PWDT: CIRS, Norfolk, VA) is a phantom material designed for water equivalence in photon beams from 0.04 MeV to 100 MeV; the material has also good mechanical properties. The present article reports the results of computed tomography (CT) imaging and dosimetric studies of PWDT to evaluate the suitability of the material in CT and therapy energy ranges.

We characterized the water equivalence of PWDT in a series of experiments in which the basic dosimetric properties of the material were determined for photon energies of 80 kVp, 100 kVp, 250 kVp, 4 MV, 6 MV, 10 MV, and 18 MV. Measured properties included the buildup and percentage depth dose curves for several field sizes, and relative dose factors as a function of field size. In addition, the PWDT phantom underwent CT imaging at beam qualities ranging from 80 kVp to 140 kVp to determine the water equivalence of the phantom in the diagnostic energy range. The dosimetric quantities measured with PWDT agreed within 1.5% of those determined in water and Solid Water (Gammex rmi, Middleton, WI). Computed tomography imaging of the phantom was found to generate Hounsfield numbers within 0.8% of those generated using water. The results suggest that PWDT material is suitable both for regular radiotherapy quality assurance measurements and for intensity‐modulated radiation therapy (IMRT) verification work. Sample IMRT verification results are presented.

PACS number: 87.53Dq

## I. INTRODUCTION

The increasing complexity in radiotherapy treatment planning and delivery warrants increased quality assurance (QA) to ensure accurate dose delivery to the patients. Dosimetric protocols recommend the use of water as the phantom for dose measurements.^(^ ^1^ ^–^ ^3^ ^)^ However, because of the tedious setup required for performing water phantom measurements, other Solid Water (Gammex rmi, Middleton, WI)–equivalent materials have been used in performing some of these measurements.

The basic requirements in phantom design call for materials with mechanical strength and resilience, and for dosimetric properties closely matched to those of water. Several Solid Water–equivalent materials are on the market, manufactured to simulate the dosimetric behavior of water as closely as possible over a wide range of energies.^(^ ^4^ ^–^ ^10^ ^)^ Of the commercially available phantom materials, polymethyl methacrylate, polystyrene, and epoxy resin are most frequently used as dosimetric phantoms. The International Commission on Radiation Units Report 44 states that, for any solid phantom to be considered water‐equivalent, it should not introduce more than 1% uncertainty to the absorbed dose. If total uncertainties reach more than 1%, appropriate correction factors have to be applied.^(11)^

A typical radiation therapy process involves scanning patients using computed tomography (CT). The resulting three‐dimensional CT image data is used in the treatment plan to calculate patient inhomogeneities. To perform true QA, it would be ideal to simulate the entire treatment process. Simulation of this kind would mean scanning a phantom using CT and then using the same phantom for radiotherapy measurements. For example, in performing patient‐specific intensity modulated radiation therapy (IMRT) verification measurements, the patient fluence is transferred to the Solid Water phantom, and a forward dose calculation is performed. The calculated dose is then compared to the point dose and to the dose distribution measured in the phantom. In this process, it is desirable that the phantom characteristics match attenuation and absorption properties in the diagnostic and therapeutic ranges alike.

Recently, CIRS (Norfolk, VA) introduced a prototype Plastic Water Diagnostic Therapy (PWDT) phantom material designed for water equivalence in photon beams from 0.04 MeV to 100 MeV. The objective of the present work was to evaluate a PWDT phantom dosimetrically for its suitability over diagnostic to radiation‐therapy energy ranges.

## II. MATERIALS AND METHODS

**Figure 1:**
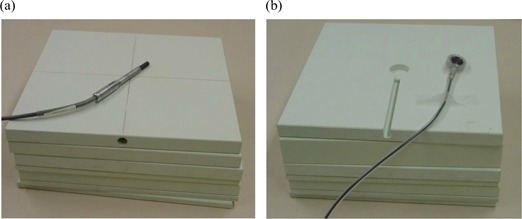
Plastic Water Diagnostic Therapy (CIRS, Norfolk, VA) phantom with (a) Farmer‐type ion chamber and (b) parallel‐plate chamber.

We characterized the water equivalence of PWDT in a series of experiments in which basic dosimetric properties were measured in both water and Plastic Water. Sheets were machined to accommodate a NACP‐02 parallel‐plate chamber (Scanditronix, IBA Dosimetry America, Bartlett, TN) and a standard 0.6 cm^3^ Farmer‐type chamber (NE2571: Nuclear Enterprises, Fairfield, NJ) as shown in Fig. 1. All measurements were performed using a Keithley 35614 electrometer (Keithley Instruments, Cleveland, OH). Measurements in water were performed using a Med‐Tec (Med‐Tec Iowa, Orange City, IA) 30×30×30‐cm phantom with a depth resolution of 0.1 mm. During in‐water measurements, the Farmer chamber was protected by a thin neoprene sheath for waterproofing.

The absorbed dose to water was calculated from ionization measurements in water, Solid Water, and Plastic Water using the American Association of Physicists in Medicine Task Group 51 (TG51) calibration protocol. The Plastic Water materials were earlier left in the treatment room for several hours to attain temperatures very close to room temperature.

### A. CT water equivalence

A GE Light Speed Plus CT scanner (GE Health Care, Waukesha, WI) was used to carry out CT measurements at various beam qualities. A 30×30×15‐cm rectangular PWDT phantom was used for scanning. An acrylic‐walled phantom of the same shape and dimensions was also scanned.

### B. Orthovoltage depth dose measurements

Orthovoltage measurements were performed on Therapax DXT300 (Therapak, Branford, CT) unit with a 10×10‐cm field size at 50 cm focus‐to‐skin distance. The NACP parallel‐plate chamber with a 1‐mm water‐equivalent entrance window was used for the measurements. Sheets of PWDT ranging from 2 mm to 4 cm in thickness were used to determine the percentage depth dose.

### C. Megavoltage depth dose measurements

Depth dose in a megavoltage beam was measured at 4 MV, 6 MV, 10 MV, and 18 MV on a Varian 2100C linear accelerator (Varian Medical Systems, Palo Alto, CA), with field sizes of 5×5 cm, 10×10 cm, and 20×20 cm at a source‐to‐surface distance of 100 cm. Depth dose measurements were performed using a Farmer chamber, and the buildup dose was measured using a parallel‐plate chamber.

### D. Comparison, using Monte Carlo simulation, of dosimetry characteristics of Solid Water, PWDT, and water

Both the Solid Water and the PWDT were scanned using a Philip Brilliance CT big‐bore scanner (Philips, Fitchbert, WI). (The GE CT scanner used earlier was not available to perform this part of the study.) Phantoms were created based on CT images. Scanned CT images of the Solid Water and PWDT phantoms were imported into an Eclipse treatment planning system (Varian Medical Systems). A pure‐water phantom (assigned a CT number of zero) was also created in the treatment planning system. All three phantoms were used for beam profile calculations with the EGS4 Monte Carlo package from the National Research Council of Canada. The plan setup used a 100 cm source‐to‐axis distance, a 95 cm source‐to‐surface distance, and a 10×10 cm field with 6‐MV photons for all three phantoms.

### E. Absolute measurements

The TG51 protocol recommends performing absolute measurements in water. We assumed that both Plastic Water and Solid Water are exactly water equivalent. Hence, our measurements were performed under conditions identical to those for water, and we used water‐phantom factors for calibration. For these measurements, we used a Capintec Model 192 electrometer and Capintac Model PR‐06‐C chamber (Capintec, Ramsey, NJ) calibrated in terms of absorbed dose to water.

### F. Additional dosimetric measurements

A Farmer chamber was used to measure relative dose factors for 5×5‐cm and 20×20‐cm fields at the nominal maximum dose for 4‐, 6‐, 10‐, and 18‐MV beams. Wedge factors were measured for 10×10‐cm and 20×20‐cm field sizes with a 6‐MV beam in water and Plastic Water at a depth of 5 cm.

Patient‐specific IMRT QA was performed using PWDT in a Varian 2100C linear accelerator with a Millennium multileaf collimator. The IMRT treatment was planned and delivered using the sliding window technique. The PWDT phantom with 0.6‐cm^3^ Farmer ion chamber was scanned in the CT scanner. These images were then transferred to the planning system. Patient fluences were transferred field‐by‐field, and a forward calculation was performed. Correction factors for dose gradients across the Farmer chamber were applied to measured points. The measurements were performed in PWDT at a depth of 5 cm and were compared to the calculations from CadPlan/Helios 6.2.7 (Varian Medical Systems).

## III. RESULTS

Before any solid material is used as a water substitute, it is very important that a comparison with measurements in water be carefully performed. The phantom materials should be carefully checked to ensure that their densities, slab thickness, and radiologic characteristics are consistent.

**Figure 2:**
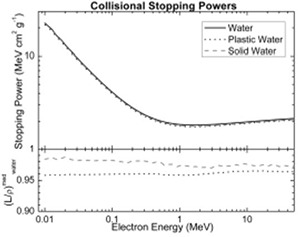
Comparison of collisional stopping powers for water, Plastic Water (CIRS, Norfolk, VA), and Solid Water (Gammex rmi, Middleton, WI).

**Figure 3:**
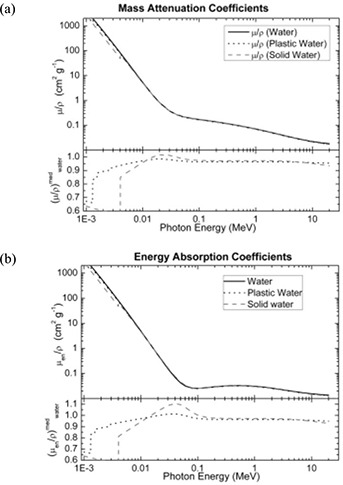
Comparison of (a) mass attenuation coefficients, and (b) energy absorption coefficients for water, Plastic Water (CIRS, Norfolk, VA), and Solid Water (Gammex rmi, Middleton, WI).

The water equivalency of PWDT was evaluated in terms of CT number, percentage depth dose, and relative dose factor. Independent experiments were carried out to compare the phantom material to water at various energies ranging from orthovoltage to megavoltage. The dosimetric properties for the newly introduced Plastic Water were calculated and compared with those of water, based on the nominal material composition. The same properties were also calculated for Solid Water, a well‐established tissue substitute. Table 1 presents the elemental composition, density, and electron density of Plastic Water. Fig. 2 plots the calculated collisional stopping power and mass collisional stopping power of water, Plastic Water, and Solid Water in the electron energy range 10 KeV – 50 MeV. Similarly, Fig. 3 plots the mass attenuation coefficient and energy absorption coefficient of photons in the energy range 10 KeV – 50 MeV.

### A. CT water equivalence

Measurements of CT number were performed at 9 regions on the slice bisecting each phantom. The mean and standard deviation of values within a circular region 3 cm^2^ about those points were measured using the software tools available on the scanner. The CT numbers were measured and compared with water to evaluate the radiation characteristics of Plastic Water for its suitability in the diagnostic range. Table 2 presents the CT numbers of water and Plastic Water measured at 80 kV−140 kV.

**Table 1:** Elemental composition of Plastic Water^a^ compared with other materials

		*Mass fraction composition (%)*		
*Element name*	*Z*	*Water*	*Plastic Water*	*Solid Water* ^b^
Hydrogen	1	11.2	7.4	8.1
Boron	5	—	2.26	—
Carbon	6	—	46.7	67.2
Nitrogen	7	—	1.56	2.4
Oxygen	8	88.8	33.52	19.9
Magnesium	12	—	6.88	—
Aluminum	13	—	1.4	—
Chlorine	17	—	0.24	0.1
Calcium	20	—	—	2.3
Weighted mean Z	—	6.6	6.43	5.96

a CIRS, Norfolk, VA.

b Gammex rmi, Middleton, WI.

**Table 2:** Comparison of computed tomography (CT) numbers between water phantom and Plastic Water^a^ phantom

*Position*	*Water phantom*		*Plastic Water phantom*		*Ratio*	
	*HU*	*σ(HU)*	*HU*	*σ(HU)*	*Rel water^b^*	*σ*
80‐kVp CT slice measurements						
Center of phantom	−2.46	32.84	0.43	15.94	1.003	0.037
Midplane of phantom, right edge	8.81	61.16	2.85	15.00	0.994	0.062
Midplane of phantom, left edge	9.87	23.20	2.87	16.20	0.993	0.028
Top of phantom, center	−1.84	32.00	−5.65	10.64	0.996	0.034
Top of phantom, right edge	5.91	62.91	−0.63	16.67	0.993	0.064
Top of phantom, left edge	4.31	26.45	−0.54	13.99	0.995	0.030
Bottom of phantom, center	5.20	30.41	3.99	14.52	0.999	0.033
Bottom of phantom, right edge	15.28	62.39	5.91	21.69	0.991	0.065
Bottom of phantom, left edge	14.70	34.61	5.56	23.63	0.991	0.041
100‐kVp CT slice measurements						
Center of phantom	2.69	22.65	−1.96	10.41	0.995	0.025
Midplane of phantom, right edge	11.09	19.23	4.69	8.23	0.994	0.021
Midplane of phantom, left edge	9.93	28.38	5.10	8.31	0.995	0.029
Top of phantom, center	0.05	17.16	−4.94	7.26	0.995	0.019
Top of phantom, right edge	10.90	15.29	1.21	7.76	0.990	0.017
Top of phantom, left edge	4.61	24.77	1.59	9.80	0.997	0.026
Bottom of phantom, center	2.74	23.78	−0.55	8.85	0.997	0.025
Bottom of phantom, right edge	11.99	19.66	8.26	11.00	0.996	0.022
Bottom of phantom, left edge	14.93	39.65	7.52	13.70	0.993	0.041
120‐kVp CT slice measurements						
Center of phantom	2.24	15.29	−2.14	6.16	0.996	0.016
Midplane of phantom, right edge	11.19	14.05	2.27	6.81	0.991	0.015
Midplane of phantom, left edge	7.82	18.23	3.74	5.26	0.996	0.019
Top of phantom, center	11.92	12.91	−6.03	5.40	0.982	0.014
Top of phantom, right edge	−0.27	12.13	−0.21	6.50	1.000	0.014
Top of phantom, left edge	4.21	14.29	0.97	5.35	0.997	0.015
Bottom of phantom, center	5.57	15.28	−0.71	6.25	0.994	0.016
Bottom of phantom, right edge	13.00	15.01	6.21	8.95	0.993	0.017
Bottom of phantom, left edge	12.50	26.95	7.36	7.28	0.995	0.027
140‐kVp CT slice measurements						
Center of phantom	3.49	13.83	−3.33	5.35	0.993	0.015
Midplane of phantom, right edge	12.83	10.04	1.64	5.20	0.989	0.011
Midplane of phantom, left edge	8.06	13.42	2.05	5.47	0.994	0.014
Top of phantom, center	−0.16	9.12	−6.86	4.13	0.993	0.010
Top of phantom, right edge	13.10	10.00	0.50	4.70	0.988	0.011
Top of phantom, left edge	5.43	14.04	0.36	5.20	0.995	0.015
Bottom of phantom, center	4.19	14.97	−1.70	5.26	0.994	0.016
Bottom of phantom, right edge	13.51	11.53	6.68	7.19	0.993	0.013
Bottom of phantom, left edge	12.92	22.11	6.78	7.31	0.994	0.023

a CIRS, Norfolk, VA

b $Rel\;Water=\frac{HU(PWDT)+1000}{HU(Water)+1000}$

HU=Hounsfield units.

### B. Orthovoltage depth dose measurements

**Figure 4:**
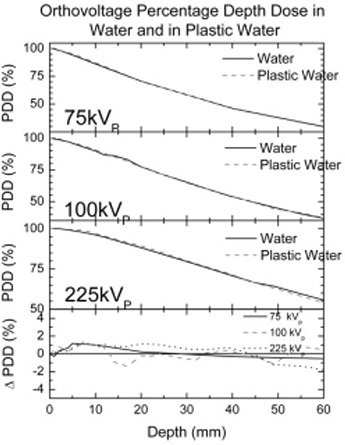
Comparison of percentage depth doses (PDDs) in orthovoltage energies.

A NACP‐02 parallel‐plate chamber was used to measure depth dose curves for beam qualities of 75 kVp, 100 kVp, and 225 kVp. Similar measurements were performed in water under identical conditions. No additional waterproofing was required for the NACP chamber, and no correction for the entrance window was applied to either measurement set. Fig. 4 shows the measured depth dose curves for the 75 kVp, 100 kVp, and 225 kVp energies for both Plastic Water and water.

### C. Megavoltage depth dose measurements

**Figure 5:**
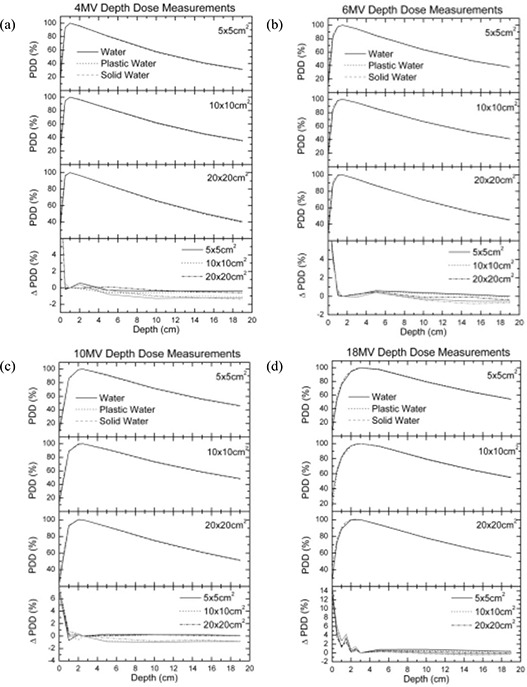
Percentage depth dose comparisons between water, Plastic Water (CIRS, Norfolk, VA), and Solid Water (Gammex rmi, Middleton, WI) for (a) 4‐MV, (b) 6‐MV, (c) 10‐MV, and (d) 18‐MV beams.

Fig. 5 shows the measured depth dose curves in Plastic Water, Solid Water, and water for the 4‐, 6‐, 10‐, 18‐MV beam qualities. A 0.6 cm^3^ Farmer chamber was used for the measurements. No corrections for the effective point of measurement were applied to the measurements.

### D. Comparison, using Monte Carlo simulation, of dosimetry characteristics of Solid Water, PWDT, and water

**Figure 6:**
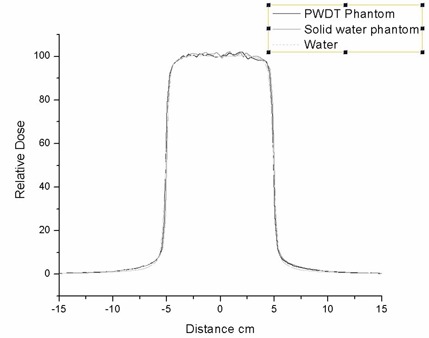
Monte Carlo comparison of a profile at 5 cm depth for water, Plastic Water (CIRS, Norfolk, VA), and Solid Water (Gammex rmi, Middleton, WI). PWDT=Plastic Water Diagnostic Therapy.

**Figure 7:**
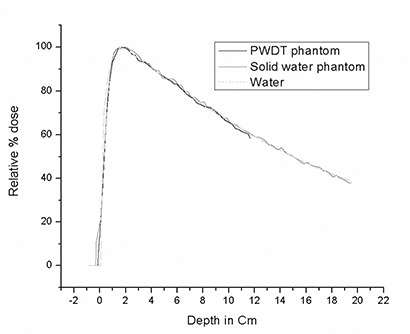
Monte Carlo comparison of percentage depth dose for water, Plastic Water (CIRS, Norfolk, VA), and Solid Water (Gammex rmi, Middleton, WI). PWDT=Plastic Water Diagnostic Therapy.

The percentage depth doses and beam profiles at a depth of 5 cm were plotted and compared, as shown in Figs. 6 and 7. The beam penumbrae defined for comparison are the regions where the isodose changes from 80% to 20% of maximum.

### E. Absolute measurements

Solid Water and the PWDT phantom were both assumed to be exactly water equivalent. Protocols similar to absolute measurements in water were performed. The $N_{d,w}^{Co-60}$ and $K_{Q}$ factors for water phantoms were used for calibration. To evaluate water equivalency, the results were compared with the water calibration.

### F. Additional dosimetric measurements

**Figure 8:**
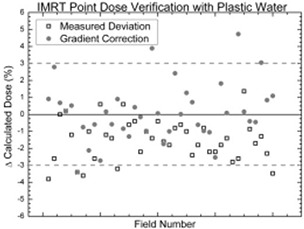
Intensity‐modulated radiation therapy (IMRT) point dose verification with Plastic Water (CIRS, Norfolk, VA).

Table 3 shows a comparison of the relative dose factors for water, Plastic Water, and Solid Water. Table 4 shows the wedge factor measurements for 6 MV. Patient‐specific IMRT QA measurements represent a patient population with cancer of the head and neck. The typical prescription is 60 Gy for the primary target and 50 Gy for the nodal chain. Typically, 9 non‐opposed fields are used for planning. Constraints to dose priorities generally result in conflicts. Such conflicts arise from the application of margins, which result in overlapping structures. A 3‐mm margin is applied to critical structures, and a 5‐mm margin is applied to targets. The priority of meeting the dose criteria is incorporated into the optimization process, either by varying the penalty associated with violations of the selected dose limits for the conflicting structures, or by creating an independent structure to represent the overlap volume, with its own dose limits and penalties. When the treatment volume approaches the skin surface, the contours are modified, and 0.5 cm is subtracted from the skin surface. Fig. 8 plots deviation from the calculated dose measured with PWDT; dotted lines show the tolerance.

**Table 3:** Comparison of relative dose factors between water, Plastic Water,^a^ and Solid Water^b^

*Energy*	*Field size*	*Water*	*Plastic Water*	*Solid Water*
4 MV	5×5 cm	0.946	0.954	0.955
	20×20 cm	1.057	1.046	1.045
6 MV	5×5 cm	0.943	0.945	0.945
	20×20 cm	1.054	1.047	1.049
10 MV	5×5 cm	0.934	0.938	0.939
	20×20 cm	1.056	1.048	1.051
18 MV	5×5 cm	0.917	0.916	0.919
	20×20 cm	1.070	1.065	1.071

a CIRS, Norfolk, VA.

b Gammex rmi, Middleton, WI.

**Table 4:** Comparison of wedge factors between water and Plastic Water^a^ Diagnostic Therapy (DT) for a 6‐MV beam

*Field size (cm)*	*Plastic Water DT*	*Water*
	15‐Degree wedge	
10×10	0.509	0.499
20×20	0.521	0.522
	45‐Degree wedge	
10×10	0.711	0.714
20×20	0.724	0.723

a CIRS, Norfolk, VA.

## IV. DISCUSSION

The mean atomic number $\bar{Z}$ is used for mixtures or compounds (or both) when the scaling parameter is used in a comparison, and it is defined as

(1) $$\bar{Z}=\frac{\sum_{i}\frac{p_{i}Z_{i}^{2}}{M_{A_{i}}}}{\sum_{i}\frac{p_{i}Z_{i}}{M_{A_{i}}},}$$

where $p_{i}$ is the mass fraction, $Z_{i}$ is the atomic number, and $M_{A_{i}}$ is the molar mass of element *i*. As compared with Solid Water, PWDT has a mean atomic number closer to that of water (Table 1). The electron density of Plastic Water is $3.345\times 10^{23}/{cm}^{3}$, and its physical density is slightly higher at $1.039\;g/{cm}^{3}$. The optimum specific gravity is 3% higher than the water it displaces. In fact, a slightly higher density of Plastic Water is required to match the electron density of water and to make the Plastic Water volumetrically equivalent to water so that the transmission of X and gamma rays through each is the same, centimeter to centimeter.^(4)^

From Fig. 2, it is evident that agreement for the collisional stopping power of Solid Water, Plastic Water, and water is generally good over the energy range studied. The mass collisional stopping power of Plastic Water between 10 keV and 50 MeV is approximately 4% less than that of water. Similarly, the Fig. 3 shows graphs of the mass attenuation coefficient and the energy absorption coefficient of photons in the energy range 10 KeV – 50Mev. Between 3 keV and 20 keV, these factors are approximately 4% lower for Plastic Water than for water. For Solid Water the factors change significantly, and agreement with water improves with increasing energy. Beyond 0.1 MeV, excellent agreement with water is observed for both Solid Water and Plastic Water. The Plastic Water—with its mass collisional stopping power, mass attenuation coefficient, energy absorption coefficient, and nominal density of $1.039\;g\;{cm}^{-3}$—provides water‐equivalent radiation transport properties within 0.5% for photons and electrons alike. Our results for water and Solid Water are in agreement with an earlier published report.^(8)^

### A. Water equivalence with CT

Phantoms are used in radio‐diagnosis primarily for patient dose measurements, assessment of image quality, commissioning, and radiation therapy treatment planning. Plastic Water exhibited a water‐equivalent CT number to within 0.7% for all qualities. The agreement of the CT numbers for Plastic Water and water shows similar absorption characteristics for low X‐ray energies. The maximum difference observed between water and Plastic Water was 17.95 CT numbers. The observed level of agreement is well within the acceptable limit.^(^ ^5^ ^,^ ^11^ ^)^

### B. Orthovoltage depth dose measurements

Fig. 4 shows depth dose curves measured for orthovoltage beam qualities of 75 kVp, 100 kVp, and 225 kVp both for Plastic Water and water. The Plastic Water dose is generally within 1% of the dose measured in water. Repeated measurements at 75 kVp indicated that depth dose results were reproducible to within 0.5% for both water and Plastic Water. The measured depth dose difference in Plastic Water for 75 kV is slightly higher than that seen with other kilovoltage energies. That finding is consistent with the small differences between PWDT and water attenuation coefficients at lower energies.

### C. Megavoltage depth dose measurements

Fig. 5 shows the measured depth dose curves in Plastic Water, Solid Water, and water for 4‐, 6‐, 10‐, and 18‐MV beam qualities. Very good agreement was observed between the water and Plastic Water measurements; it was within 0.5% of the depth dose measured in water beyond the maximum dose. The Plastic Water depth dose measurements were closer to water than were the Solid Water dose measurements. However, both phantoms had a high degree of agreement with water, but with smaller variation between them. Such variation is expected because of the difference in the ratio of the mean restricted mass collisional stopping powers and the mean mass energy absorption coefficients. It is also important to note that, when making measurements in Plastic Water phantom material, the temperature in the chamber cavity may be different from the temperature of the air in the room by several degrees. The practice of assuming that the air in the chamber inside Plastic Water phantom material is at room temperature can easily introduce a dose discrepancy of up to 1%.^(12)^ Although the solid phantom materials were earlier left in the treatment room for several hours, we have observed that the temperature of the treatment room changes over the course of the treatment day. That temperature variation is expected to have some effect, because the air cavities in the solid phantoms take a long time to reach equilibrium.

### D. Comparison, using Monte Carlo simulation, of dosimetry characteristics of Solid Water, PWDT, and water

In the Monte Carlo–simulated data, the depth of maximum dose for PWDT was slightly less (closer to the surface) than it was for Solid Water, but it was closer to that for water. In the buildup region, the percentage depth dose from PWDT had better agreement with water than did the Solid Water. In general, the Monte Carlo calculation for Plastic Water agreed well with the water calculations. The agreement of Plastic Water with water in the buildup region supports the fact that, as compared with Solid Water, Plastic Water has good water properties at low energies.

### E. Absolute measurements

The TG51 absolute dose measured using a 6‐MV beam for PWDT was 1.004 cGy per monitor unit (MU), and the dose measured with water was 1.01 cGy/MU. Similarly, the absolute dose measured with PWDT at 18 MV was 1.007 cGy/MU, and the water measurement was 1.012 cGy/MU. The measured dose with PWDT was 0.6% lower than that with water, and the Solid Water was lower by 1.5%. These results show that Plastic Water is closer to water than Solid Water is. The lower dose recorded with Plastic Water and Solid Water could be attributed to relatively lower mass collisional stopping power, mass attenuation coefficient, and energy absorption coefficient in solid phantoms as compared with water. Such a measurement may be useful when a practical need exists to put dose measurements using Plastic Water or solid phantoms on an absolute basis. The minor differences can be scaled and corrected for absolute dose measurements. Therefore, Plastic Water can potentially be used for performing absolute calibration once appropriate correction factors are applied.

### F. Additional dosimetric measurements

The relative dose factor for 4‐, 6‐, 10‐, and 18‐MV photon beams measured with water, Solid Water, and Plastic Water show good agreement (Table 3). Plastic Water and Solid Water both showed the same level of agreement in each instance, with measurements agreeing within 1% of water measurements. Similarly, good agreement on wedge factors in a 6‐MV beam was obtained for 15‐degree and 45‐degree wedges for water and Plastic Water alike.

Fig. 6 summarizes IMRT point dose verification measurements for 40 different IMRT fields, which can be seen to be within the tolerance of the calculated value. This level of agreement was achieved by modeling the Farmer chamber in the treatment planning system. From the dose–volume histogram of the modeled ion chamber, the mean dose is used as a correction factor. The variation of the measurements from the calculated point doses was consistent within the expected nominal range of ±3% for such fields.

The measured results in Solid Water and Plastic Water phantoms are specific to the particular batches of the solid phantom material. Manufacturing variations in the phantom materials may lead to variations in their physical properties, such as physical density. Such variations can result in dosimetric variations, and 3%−4% variations have been reported in the literature.^(10)^ In addition, dosimetric implications may arise from non‐uniform response in low‐energy X‐rays, such as those used in diagnostic imaging.

## V. CONCLUSIONS

The radiation characteristics of Plastic Water were found to be very close to those of water, not only in the radiotherapy beam range, but also in the range of diagnostic X‐rays. The mass collisional stopping power, mass attenuation coefficient, and energy absorption coefficient of photons in the energy range 10 KeV – 50 Mev math for Plastic Water agreed to within 4% of those parameters for water. The CT numbers of Plastic Water were within 0.7% of the CT numbers for water for all beam qualities. In the orthovoltage range, the measured percentage depth dose of PWDT was within 1% of that of water. This level of water‐equivalence agreement of PWDT in the kilovoltage range makes the material suitable for use in geometric dosimetry phantoms for CT imaging. The megavoltage dosimetric measurements agreed in general to within 0.5% of water measurements, a finding supported by the Monte Carlo–simulated data. The IMRT patient‐specific QA measurements confirmed that the PWDT materials are a good substitute for water.

We showed that PWDT provides good dosimetric accuracy, comparable with that of other water substitutes, while exhibiting good mechanical strength and resilience.
